# Expanded knowledge of cell-free DNA biology: potential to broaden the clinical utility

**DOI:** 10.20517/evcna.2022.21

**Published:** 2022-08-10

**Authors:** Huiwen Che, Kate Stanley, Tatjana Jatsenko, Bernard Thienpont, Joris Robert Vermeesch

**Affiliations:** ^1^Department of Human Genetics, Laboratory for Cytogenetics and Genome Research, KU Leuven, Leuven 3000, Belgium.; ^2^Department of Human Genetics, Laboratory for Functional Epigenetics, KU Leuven, Leuven 3000, Belgium.; ^3^Center for Human Genetics, University Hospitals Leuven, Leuven 3000, Belgium.

**Keywords:** Cell-free DNA, liquid biopsy, biomarker

## Abstract

Noninvasive sampling of an individual’s body fluids is an easy means to capture circulating cell-free DNA (cfDNA). These small fragments of DNA carry information on the contributing cell’s genome, epigenome, and nuclease content. Analysis of cfDNA for the assessment of genetic risk has already revolutionized clinical practice, and a compendium of increasingly higher-resolution approaches based on epigenetic and fragmentomic cfDNA signatures continues to expand. Profiling cfDNA has unlocked a wealth of molecular information that can be translated to the clinic. This review covers the biological characteristics of cfDNA, recent advances in liquid biopsy and the clinical utility of cfDNA.

## INTRODUCTION

Invasive diagnostic tests, such as tissue biopsies, are limited by procedure-associated risks and sometimes sampling difficulties. There is a strong need for noninvasive or minimally invasive biomarkers that do not rely on targeted sampling and expedite the often lengthy process of identifying disease. Cell-free DNA (cfDNA) has emerged as a vital biomarker for detecting and monitoring disease. Assessment of cfDNA has provided new opportunities to noninvasively obtain information from source tissues of interest. The use of cfDNA has been explored in different fields and implemented to varying extents. In obstetrics, for example, multiple large-scale clinical studies have successfully used cfDNA for prenatal screening of certain genetic conditions, underscoring the clinical value of cfDNA analysis. Specifically, common fetal aneuploidy screening is of high clinical relevance for pregnancy management. In oncology, the use of cfDNA for early cancer detection, prognosis, and treatment is under particularly extensive investigation. Moreover, there is a rising interest in broadening the diagnostic scope of cfDNA both in terms of disease and methodology. In this review, we have summarized current understandings of cfDNA and the clinical utility of cfDNA in different medical fields.

### Cell-free DNA

**Figure 1:**
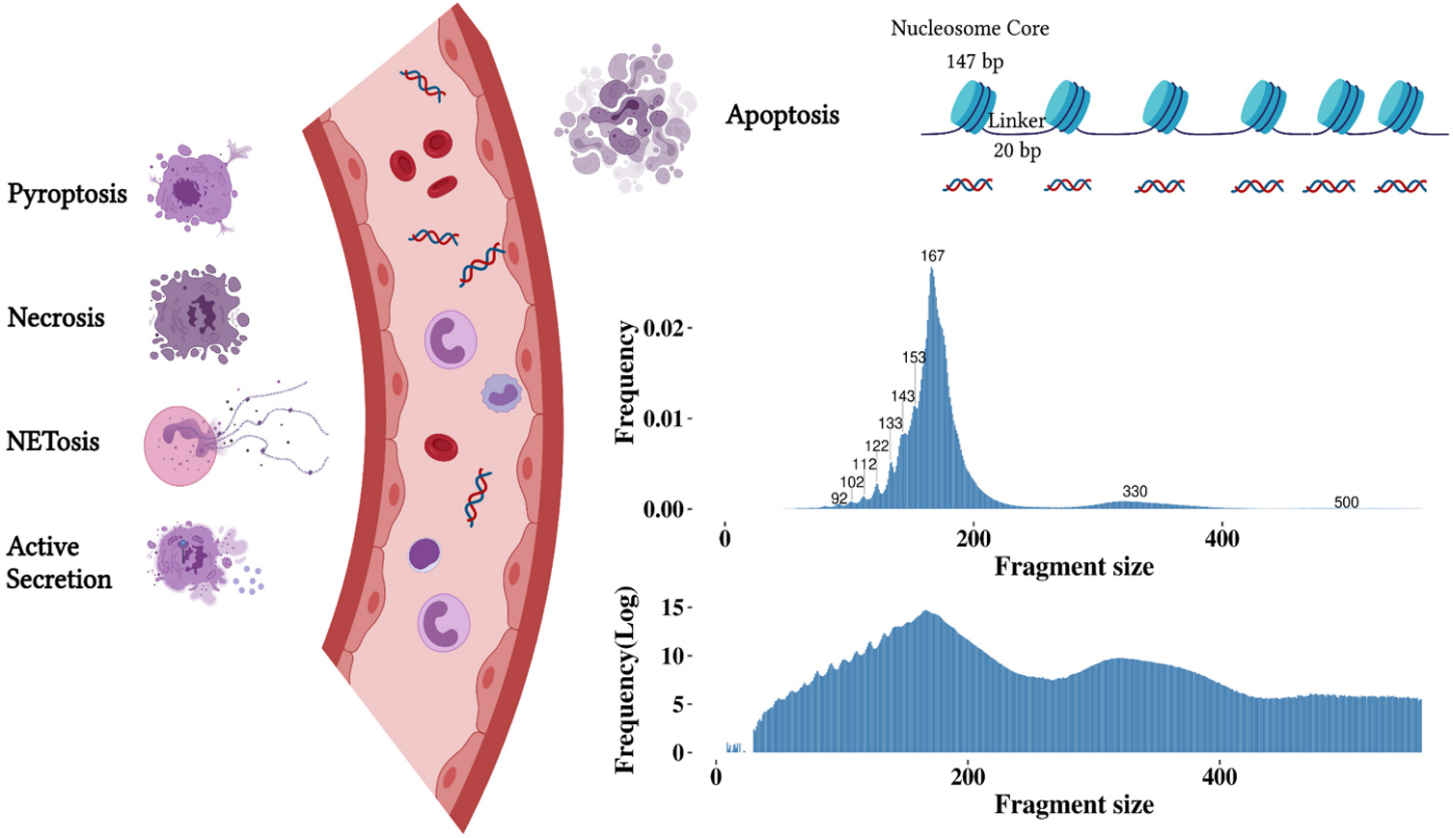
Illustration of cfDNA release mechanisms and the resulting fragment size (in bp) from apoptosis. Different forms of cfDNA release have been suggested, these primarily include apoptosis, pyroptosis, necrosis, the release of neutrophil extracellular traps (NETosis) and active secretion. Apoptosis is considered the primary source of cfDNA, resulting in non-random fragmentation. Other mechanisms of cell death, such as, pyroptosis that is inflammatory-regulated, necrosis that occurs due to accidental cell death, and NETosis that is neutrophil-specific, can also contribute to the release of cfDNA. In addition to cell death, cfDNA might be derived from active cellular secretions. The fragment size distribution of cfDNA shows peaks in sizes below 167 bp and peaks corresponding to nucleosome units. The log-transformed fragment size distribution demonstrates the di- and tri-nucleosome peak.

Cell-free DNA (cfDNA) is ubiquitous in human body fluids, including blood, urine, cerebrospinal fluid, sputum, ascites and pleural effusion^[1,2]^. Serum and plasma cfDNA have been extensively studied. It is generally accepted that cfDNA in the blood of healthy individuals is derived primarily from apoptotic hematopoietic cells^[3,4]^. Hematopoietic cell lineages have a fast turnover rate and short half-life^[5]^. In line with this, studies have shown that the main cellular origin of cfDNA is from hematopoietic cells^[3,6]^. The fragment size distribution of cfDNA is referred to as the apoptotic ladder because it matches the progression of nucleosome units with successive peaks at ~167 bp corresponding to the length of DNA wrapped around a mononucleosome (147 bp) plus the linker regions (20 bp)^[7,8]^, at ~330 bp for the di-nucleosome and at ~500 bp for the tri-nucleosome^[9-11]^. DNA fragments below 167 bp exhibit a series of smaller peaks at a periodicity of ~10 bp, likely reflecting the DNA helical repeat and cleavage at groove regions where DNA bends sharply around the nucleosome^[12,13] ^[Figure 1]. Other types of cfDNA release mechanisms have also been suggested under certain pathological conditions^[14]^. For instance, the presence of DNA fragments over 10 kilobases in cancer patients may be indicative of necrosis^[10]^. NETosis, pyroptosis and active secretion have also been proposed as putative sources of cfDNA^[15,16]^. In addition to cfDNA release, studies have revealed impaired cfDNA clearance in a number of (patho)physiological states^[17-19]^. Extracellular nucleases from the deoxyribonuclease (DNase) family are capable of digesting internucleosomal linker regions and enzymatically clearing free and protein-bound DNA^[20,21]^. CfDNA is then eliminated from circulation through organs, such as the liver, spleen, and kidney^[22]^. Abnormal DNase activity and insufficient clearance of cellular debris have been associated with elevated cfDNA concentration in patients with autoimmune diseases^[23,24]^ and cancer^[25]^. Altogether, cfDNA reflects a heterogeneous, complex and dynamic landscape of dying cells in an individual and holds great promise for noninvasive molecular testing.

### Cell-free fetal DNA

**Figure 2:**
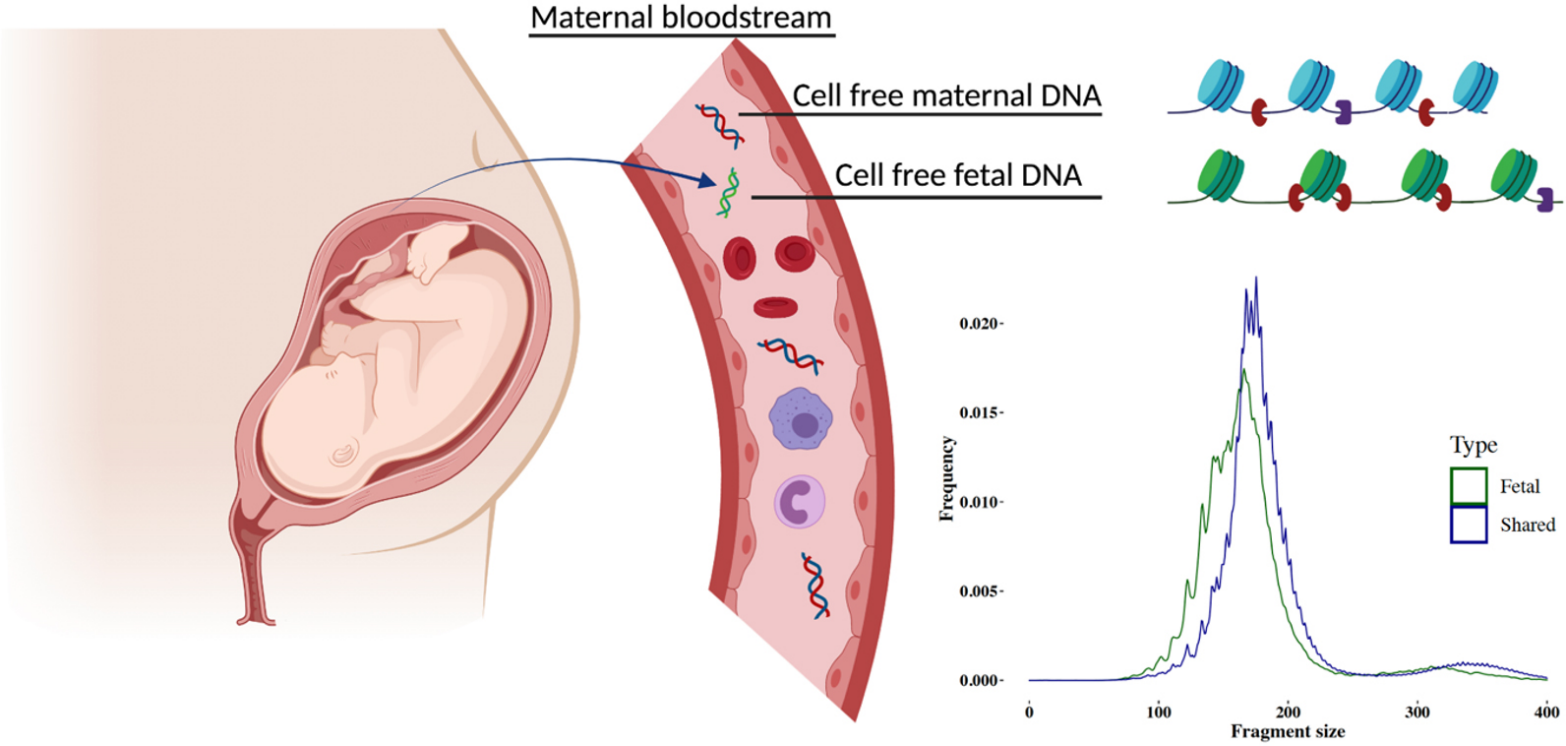
Illustration of maternal plasma DNA and an example of the fragment size (in bp) distribution of fetal-specific DNA (green) and shared (maternal and fetal; blue) DNA. cffDNA has shifted size distribution, being overall shorter than the maternal counterparts.

During pregnancy, fetal DNA fragments are detected in the maternal plasma DNA population. Y chromosomal DNA fragments from a male fetus were observed in the cfDNA of a pregnant individual as early as 1997^[26]^. It is well-recognized that cell-free fetal DNA (cffDNA) mainly originates from placental trophoblast cells. This conclusion has been drawn from the observation that cffDNA was detectable in anembryonic pregnancies and at early gestational weeks before fetal organ development^[27,28]^. Moreover, cffDNA shares methylation signatures common to trophoblast cells^[29-31]^ and, in the event of confined placental mosaicism, cffDNA reflects the genotype of the placenta rather than the fetus proper^[32,33]^. Though the cffDNA concentration increases as gestation advances, the placental contribution to the pool of cfDNA in maternal plasma, often described as the fetal fraction (FF), remains a minor fraction, usually 10%-20% throughout the first and second trimester^[34]^. The release of cffDNA is tightly linked to placental morphogenesis. Consequently, placental dysfunction can directly affect circulating cffDNA levels. For example, in pregnancies with preeclampsia or at risk of developing preeclampsia, elevated cffDNA levels have been reported^[35-37]^. Maternal conditions, such as obesity, can lead to lower FF due to higher maternal contributions^[38,39]^. CffDNA is rapidly removed from maternal circulation after delivery and the estimated half-life of cffDNA clearance is about 1 h^[40]^. Fragment size of cffDNA is generally shorter than the maternal cfDNA and peaks at around 143 bp^[9]^ [Figure 2]. The shorter size of cffDNA may be related to nucleosome organization and chromatin accessibility in placental tissue^[41]^. Assay for Transposase-Accessible Chromatin (ATAC) sequencing has revealed that chromatin is more accessible in placental tissue and may indicate that DNA is more likely to be cut close to nucleosome cores resulting in shorter fragments^[41]^. The basis for investigating the fetal genotype noninvasively is that the entire placental genome is present in the maternal plasma. Lo *et al.* showed that the proportion of cffDNA in maternal plasma is constant across the genome^[9]^. The coverage of cffDNA fragments across the genome can, however, fluctuate from region to region, potentially indicative of nucleosomal degradation patterns in the tissue of origin^[42,43]^.

### Circulating tumor DNA

**Figure 3:**
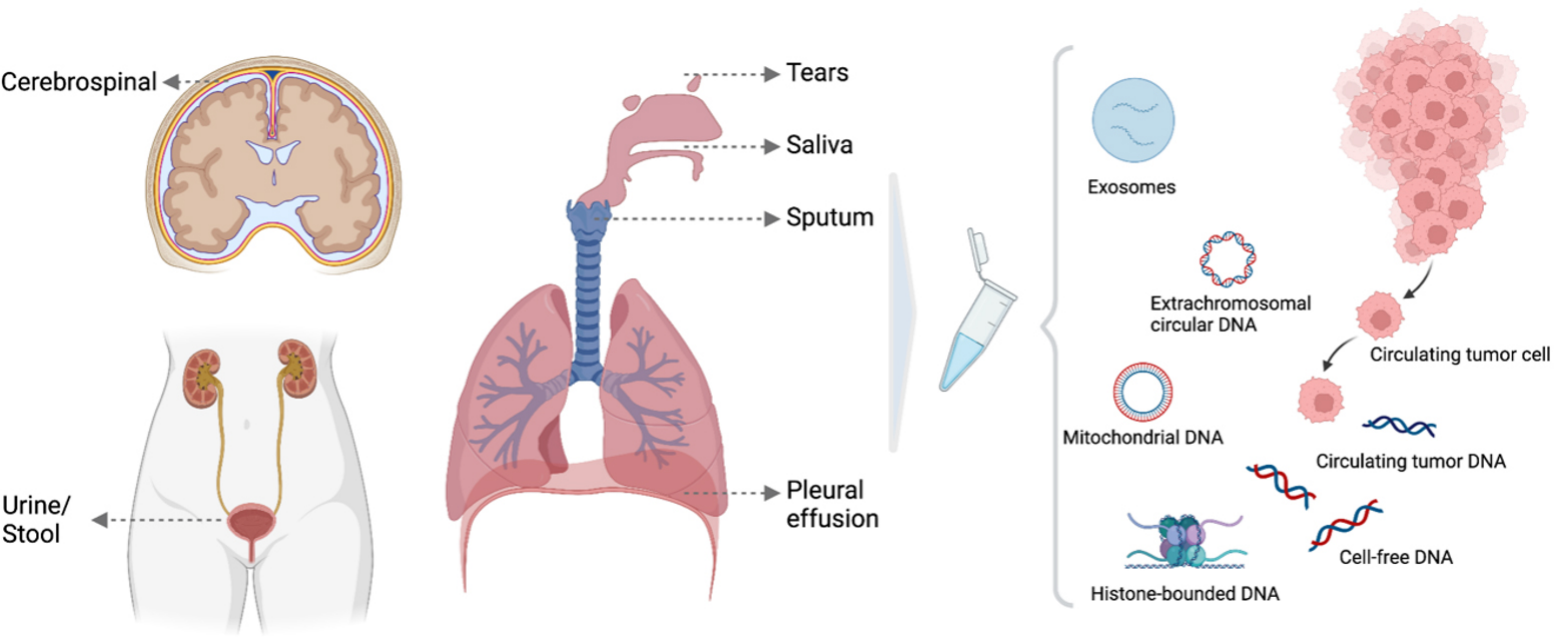
Illustration of ctDNA derived from other body fluids other than blood. Different forms of markers present in biological fluids, including circulating tumor cells, circulating cell-free DNA and circulating tumor DNA, histone-bounded DNA, exosomes, extrachromosomal circular DNA and mitochondrial DNA.

The surge of interest in cfDNA was not only sparked by the discovery of cffDNA, but also by the discovery of circulating tumor DNA (ctDNA) in cancer patients decades ago^[44-46]^. Studies have shown that genetic alterations observed in ctDNA reflect copy number aberrations and somatic mutations in the primary tumor or multiregional tumor biopsies^[47-49]^. Analysis of nucleosome maps of plasma cfDNA^[50]^ and methylation signatures^[4]^ in cancer patients have provided further evidence that ctDNA is derived from the malignant cells. It has also been postulated that non-malignant cells that are part of the tumor microenvironment, such as stromal, endothelial, lymphocytes and other immune cells, may contribute to plasma DNA of cancer patients^[1,51]^. The amount of ctDNA in the circulation, also termed tumor fraction, is likely to be associated with tumor mass/burden, cell turnover and disease stage. Increased amounts of ctDNA were found in cancer patients with advanced stage compared to early stage of diseases^[52]^. Changes in tumor fractions also reflect treatment responses over the course of therapy^[53,54]^. Nevertheless, tumor fraction in plasma DNA can remain very low even in metastatic diseases, with ctDNA levels varying across different tumor types and even subtypes^[52,55,56]^. The anatomical location of the tumor can also influence the amount of ctDNA that is shed into body fluid [Figure 3]. As an example, in patients with bladder cancer, ctDNA is more easily detected in urine compared to in blood^[57,58]^. Lastly, recent works using paired-end sequencing suggest that malignant cell-derived DNA fragments are in general shorter than those of non-malignant DNA^[50,59]^.

### Strategies to analyze plasma cfDNA

Quantitative and qualitative analyses have been developed to interrogate cfDNA. Pre-analytical factors can affect properties of cfDNA. Studies have evaluated the impact of variables, including blood collection, processing, plasma isolation and storage. Recommendations and development of standardized protocols to enhance test performance are advised^[60-62]^. Quantitative assessment usually involves the extraction of DNA from plasma or other fluids for concentration quantification. CfDNA concentration as a biomarker has been investigated widely in prenatal, cancer and autoimmune disease applications^[63-65]^, as levels of total cfDNA are known to increase under certain conditions and fluctuate in accordance with disease state. In addition to levels of total cfDNA, levels of cffDNA and ctDNA in the plasma of pregnant women and cancer patients, respectively, may also differ from those of controls. By making use of Y chromosome- or placental-specific markers, studies have shown that cffDNA levels in complicated pregnancies appear to be higher^[36,66]^. Likewise, ctDNA level, measured by a mutation template, has been explored as a biomarker for cancer detection and treatment response^[52,67]^. 

**Figure 4:**
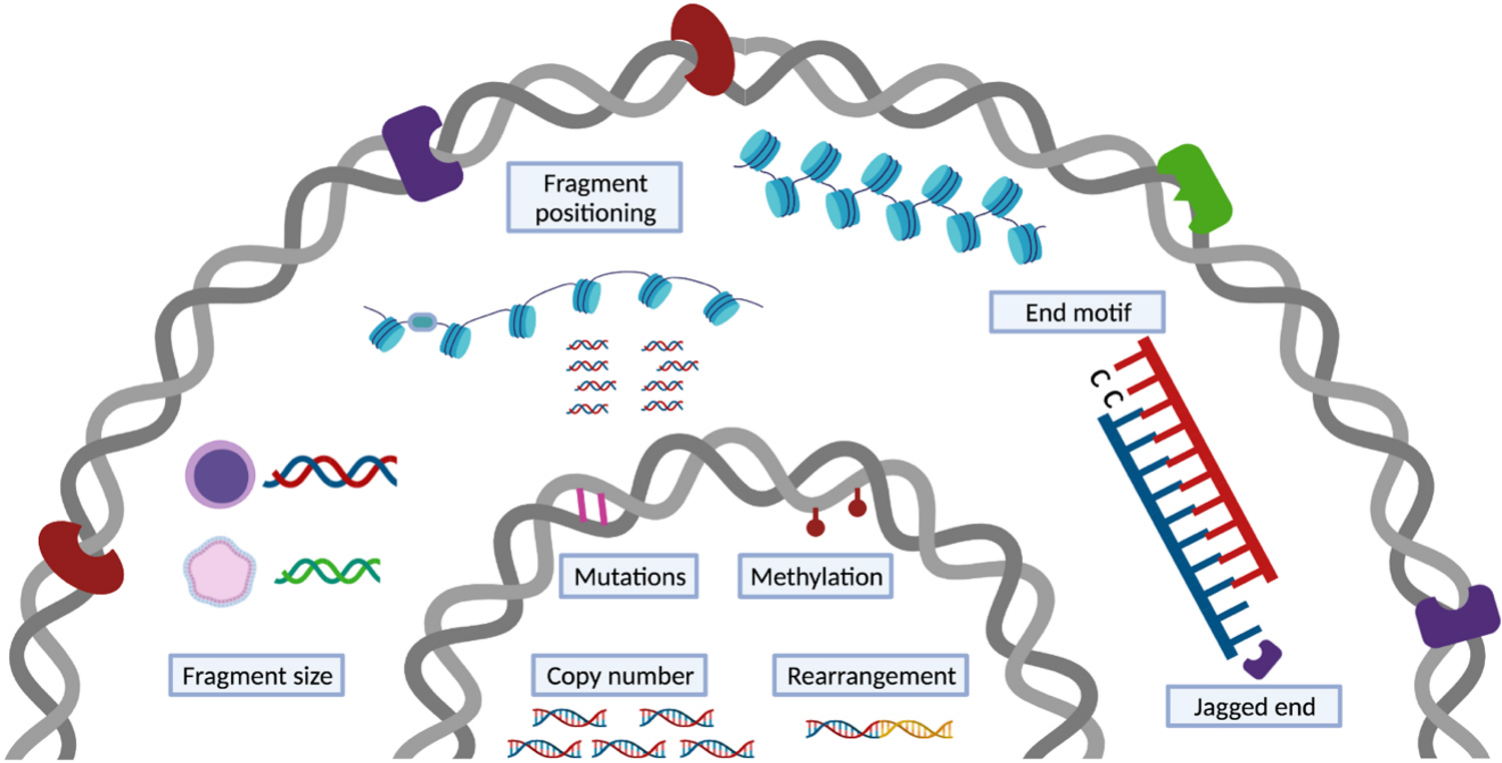
cfDNA carries (epi)genetic and fragmentation signatures. A catalog of genetic and non-genetic signatures that might be used for disease detection. The genetic signatures, including copy number changes, single nucleotide variations, and rearrangements, and epigenetic signatures, mainly methylation, are vastly investigated and used in clinical applications. The non-genetic changes that encompass several dimensions of characteristics resulted from cfDNA release mechanisms are under exploration.

While the abundance of total cfDNA and its sub fractions can indicate the presence of (patho)physiological states, a more comprehensive view is obtained by qualitative analyses. Those include the detection of genetic variations, methylation signatures and fragmentation patterns. Next generation sequencing (NGS) technologies have enabled analysis at unprecedented throughput and resolution. The presence of placenta- or tumor-derived DNA in cfDNA pools can be determined through the detection of genetic and epigenetic variations [Figure 4]. The limited absolute number of DNA molecules and low cffDNA or ctDNA concentration against high maternal or non-malignant backgrounds in plasma samples directly affects the signal identification. Though the biological limitations pose obstacles to robust detection of variations, approaches have been developed to address these challenges. One of the first methods that demonstrated sensitive detection of fetal aneuploidies relied on massive parallel sequencing to profile genomic representations of cfDNA^[68,69]^. Quantifying the statistical deviations of a profile from an external reference profile comprised of normal controls or the deviation of one region from other regions within the same sample allows robust detection of common fetal aneuploidies - an approach that has been validated by multiple large-scale clinical studies^[70-72]^. The strategy has been shown to capture copy number aberrations within ctDNA as well^[73,74]^. Deep sequencing of single-nucleotide polymorphisms and, in the case of cancer, somatic point mutations, can also be performed for the quantification of cff- and ct-DNA, respectively. In cancer studies, to compensate for the low number of ctDNA source molecules, larger numbers of mutations are tested to improve sensitivity^[75]^. Using unique molecular identifiers and analytical error corrections can further facilitate variant or mutation detection^[76-78]^. A recently developed approach that leverages two or more mutations occurring on the same strand of DNA has further pushed the detection limit. Using a personalized panel, the approach allowed identification of variants with a tumor fraction as low as 1/1,000,000^[79]^. Another type of genetic alteration - rearrangements - have been tested as part of NGS solutions specific for ctDNA detection, and a high concordance between cfDNA and tumor tissue was obtained for selected gene fusion analyses^[80,81]^.

Epigenetic approaches have been exploited extensively in recent years, among which, DNA methylation has been widely adopted for cfDNA analysis in cancer. Shortly after the discovery of tumor- and fetal-derived DNA in plasma samples, tumor and fetal specific methylation patterns were also observed in cfDNA^[82,83]^. Early in disease development, DNA methylation can exhibit divergent patterns across different tissues/cells^[4,6]^. Methylation changes thus offer the potential to predict and monitor disease states. Early investigative approaches were limited to DNA methylation marks on individual genes^[29,82,84]^, but have expanded to genome-wide assays^[85,86]^ aimed at improving sensitivity and specificity. For example, approaches that leverage multiple informative CpG markers^[87]^ or methylation haplotype blocks that exhibit highly coordinated methylation across consecutive CpG sites^[88]^ have been developed. The main method to analyze cytosine methylation at single nucleotide resolution is bisulfite conversion. The method makes use of sodium bisulfite treatment, which converts unmethylated cytosine residues to uracil and leaves 5-methylcytosine (5mC) unaffected. This approach has a major drawback in that it causes degradation of the DNA. To overcome this limitation, new methods for methylation analysis are being developed. Such methods include cell-free methylated DNA immunoprecipitation and high-throughput sequencing (cfMeDIP-seq)^[89]^, enzymatic methyl-seq (EM-Seq)^[90,91]^, and ten-eleven translocation (TET)-assisted pyridine borane sequencing (TAPS)^[92]^, which have all been successfully applied for methylation analysis in cfDNA. Furthermore, third-generation sequencing, including single-molecule real-time sequencing by Pacific Biosciences and nanopore sequencing by Oxford Nanopore Technologies, has opened new avenues for cfDNA analysis, enabling simultaneous real-time analysis of DNA sequence as well as nucleotide modifications. Though sequencing fragmented cfDNA is still challenging, proof-of-concept studies have demonstrated the potential of using these new technologies for the development of noninvasive disease management tools^[93]^. Beyond methylation patterns, chromatin immunoprecipitation and sequencing (ChIP-seq) has been developed to investigate histone modifications, allowing cell-of-origin determination and disease-specific transcriptional programs to be identified^[94]^.

In addition to genetic and epigenetic changes, various analyses that rely on cfDNA fragmentation patterns have come into view. Fragmentation of cfDNA is non-random. It is mechanically related to modes of cell death and nuclease cleavage, and cellularly associated with the nucleosome organization, chromatin structure and gene expression of the tissue of origin. Fragmentation-based signatures or fragmentomics therefore reflect multiple processes that may provide additional markers for clinical use. CfDNA fragment size is one the most thoroughly assessed fragmentation signatures. It has long been recognized from the fragments’ ladder-like patterns that cfDNA bears a signature of caspase-dependent apoptosis^[7,10]^. More recent data has shown size differences between contributing tissues. Even though the exact cause of size differences between fragments from different tissues remains to be clarified, fragment size patterns have been leveraged as a single metric or as part of a series of informative markers. CfDNA fragment size was demonstrated as an approach for fetal fraction estimation, fetal aneuploidies, and tumor detection^[95,96]^. Shorter fragments have been selected *in vitro* or *in silico* in order to enrich for DNA that is more likely to be derived from a tissue of interest. Enriching for fragments from a specific tissue based on the fragment size can also enhance genetic and epigenetic signals in downstream qualitative analyses^[97-99]^. 

Beyond size profiling, the genomic location of cfDNA fragments reflects nuclear DNA architecture and gene expression in source tissues and can be used as the basis of tissue-of-origin analyses. Fragment position is known to reflect nucleosome protection of DNA from digestion. Nucleosome linker regions are more likely to be cleaved and thus play a role in preferred cfDNA fragment ends. Based on this knowledge, nucleosome occupancy maps were constructed by identifying regions with pile ups of fragment endpoints and therefore predicted to be nucleosome-free. These analyses demonstrated periodic coverage densities and nucleosome spacing that varied with chromatin state and gene activity^[43,50]^. Therefore, the contribution of different cell types can be ranked based on the correlation of their gene activity with the observed nucleosome spacing in gene bodies^[47,100]^. Different preferred end sites were found in fragments of fetal and maternal origin, and in those of malignant and non-malignant origin in liver cancer^[41]^. When quantifying the preferred end signature in open chromatin regions, the measure of pooled density differences of directional DNA ends could be used to trace the tissue of origin of cfDNA fragments^[101]^. In addition to preferred end sites, other fragmentation characteristics include end motifs and jagged ends. At the nucleotide resolution, the 5’ fragment 4-mer end motif was found to hold tissue-of-origin information as well^[102]^. A catalog of end motifs have altered frequencies in cancer, pregnant and transplantation subjects when compared with healthy controls and the diversity of the end motifs present in cfDNA has be used in cancer detection^[102]^. In cancer patients, these differences were ascribed to a deficiency in the DNASE1L3 nuclease^[103,104]^. Lastly, Jiang *et al.* explored the single-stranded overhangs by methylation analysis to infer deliberate cleavage events^[105]^. DNA end repair could introduce unmethylated cytosine using CpG sites or methylated cytosine using CH sites to newly synthesized ends. Interrogating the methylation levels along the DNA fragments could provide traces of enzymatic cutting of cfDNA fragments and their tissue-specific degradation patterns. The presence of such jagged or protruding ends corroborates the non-random fragments generated by endonucleases in plasma DNA and these jagged ends may be used as an indicator for relative activities of the molecular scissors, for example DNASE1 and DNASE1L3^[106,107]^.

### Clinical diagnostic applications of cfDNA

#### Noninvasive prenatal screening for aneuploidies

The landmark finding of cffDNA in maternal plasma paved the way for the clinical translation and commercialization of cfDNA analysis for the noninvasive prenatal screening (NIPS) of common aneuploidies. NIPS is offered to pregnant women at high risk of carrying a fetus with a common trisomy 21, 18, or 13 in many countries. In Belgium and the Netherlands, NIPS is offered as a first-tier test to all pregnant women. In a recent analysis of NIPS performance in the general obstetric population, sensitivities and specificities were 98.91% and 99.98% for trisomy 21, 97.47% and 99.99% for trisomy 18, and 100% and 99.97% for trisomy 13, respectively. The positive predictive values (PPVs) were 92.39% for trisomy 21, 84.62% for trisomy 18 and 43.95% for trisomy 13^[108]^. NIPS for common aneuploidies has demonstrated superior performance. In addition to the test performance. Not only does it perform well, NIPS is usually done starting from 10 weeks of pregnancy, which is earlier than or within the same time frame as invasive and first trimester combined tests. The widespread introduction of NIPS into routine prenatal care has raised concerns about disability rights, equitable access to the test, and reproductive choices^[109]^. Despite these ethical concerns and ongoing debate about the clinical utility, it is likely that NIPS will continue to be adopted as a first-tier screening test.

Expanded NIPS that offers additional indications for rare autosomal trisomies (RATs) and structural anomalies is available, though PPVs (ranging from 0 to 21%) for detecting RATs or structural anomalies are lower than those of the common trisomies^[110]^. With that, the PPVs of expanded NIPS is considerably higher than the First Trimester Combined Test. While the clinical implementation of expanded NIPS is still under debate, the prevalence of RATs and structural anomalies is as high as those of trisomy 18 and 13^[111]^, which has motivated larger prospective studies to shed light on the clinical significance. Constitutive RATs rarely result in a viable pregnancy, however, in some cases mosaic RATs can result in adverse pregnancy outcomes, including growth impairment^[112,113]^. Determining the impact of these events early on in gestation could have clinical value and could support the adoption of expanded NIPS. False or inconclusive results can be the result of multiple biological factors, including confined placental mosaicism, the presence of a vanishing twin, maternal health conditions, and medication^[114,115]^. Continuous efforts have been made to investigate the clinical utility of the secondary findings relevant to maternal health, leading to updates in clinical management recommendations in NIPS^[114]^.

#### Noninvasive prenatal screening for monogenic disorders

**Figure 5:**
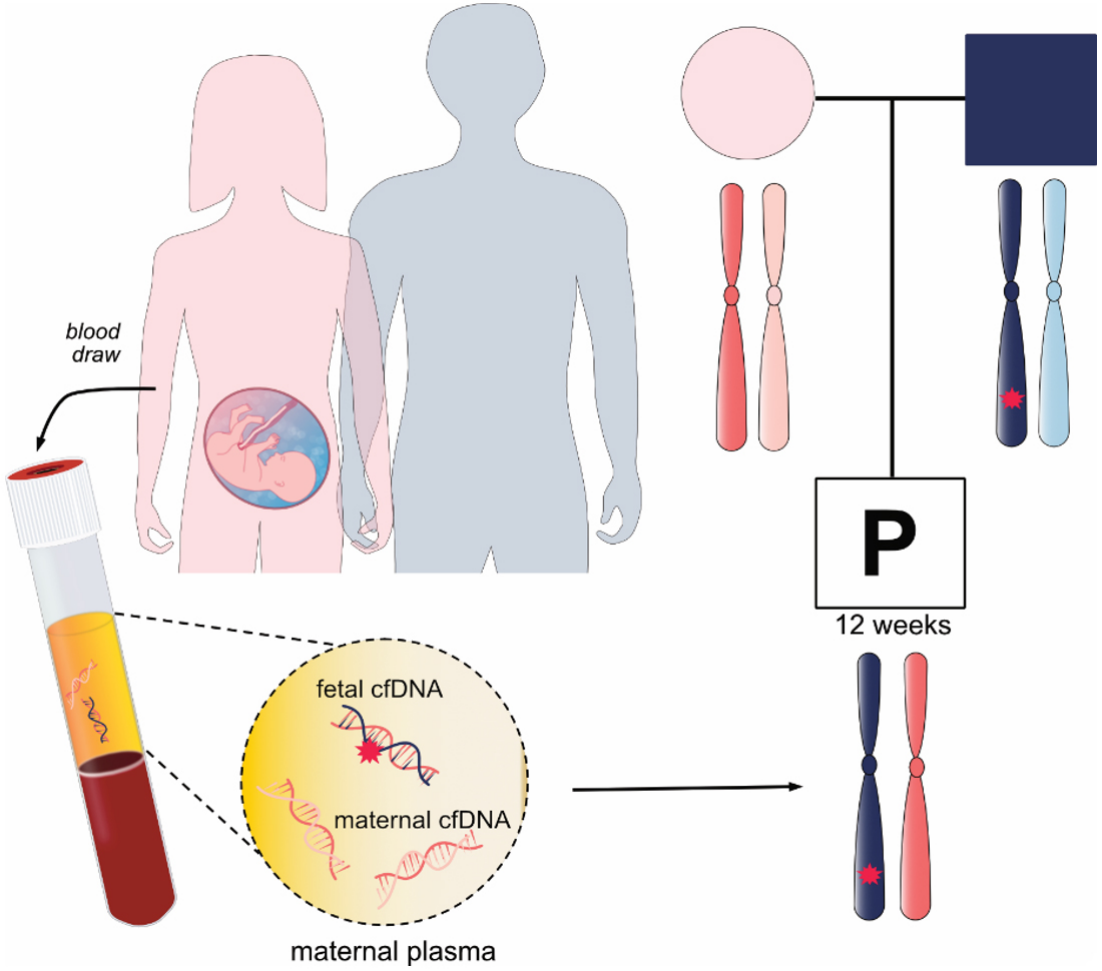
Noninvasive prenatal screening for monogenic disorders or noninvasive prenatal diagnosis. A family in which the father affected with an autosomal dominant disease opts for noninvasive prenatal diagnosis. Maternal plasma is taken to examine whether the fetus inherits the disease-associated allele.

Noninvasive prenatal screening for monogenic diseases (NIPS-M) or noninvasive prenatal diagnosis is gaining interest in the clinic. Complimentary to NIPS for aneuploidies, NIPS-M can determine the mutational status of a fetus at high risk for a single-gene disorder using maternal plasma cfDNA analysis [Figure 5]. The test is most often offered for *de novo* or paternally inherited mutation diagnosis by counting the dosage of mutant and wild-type alleles or by reconstructing haplotypes. The UK National Health Service Laboratory clinically implemented NIPS-M for inherited conditions (e.g., achondroplasia, thanatophoric dysplasia and cystic fibrosis)^[116,117]^. A recent clinical study reported robust performance in detecting dominant monogenic diseases^[78]^. Though detection of recessive or maternally inherited conditions mostly remain in the research phase, a number of technologies that readily detect a range of dominant and recessive diseases have been developed^[118-120]^. NIPS-M has been more slowly implemented in the clinic compared to NIPS for aneuploidy, which may be due to the technical difficulty of designing bespoke tests for each family based on their genetic information.

#### Liquid biopsy in cancer

**Figure 6:**
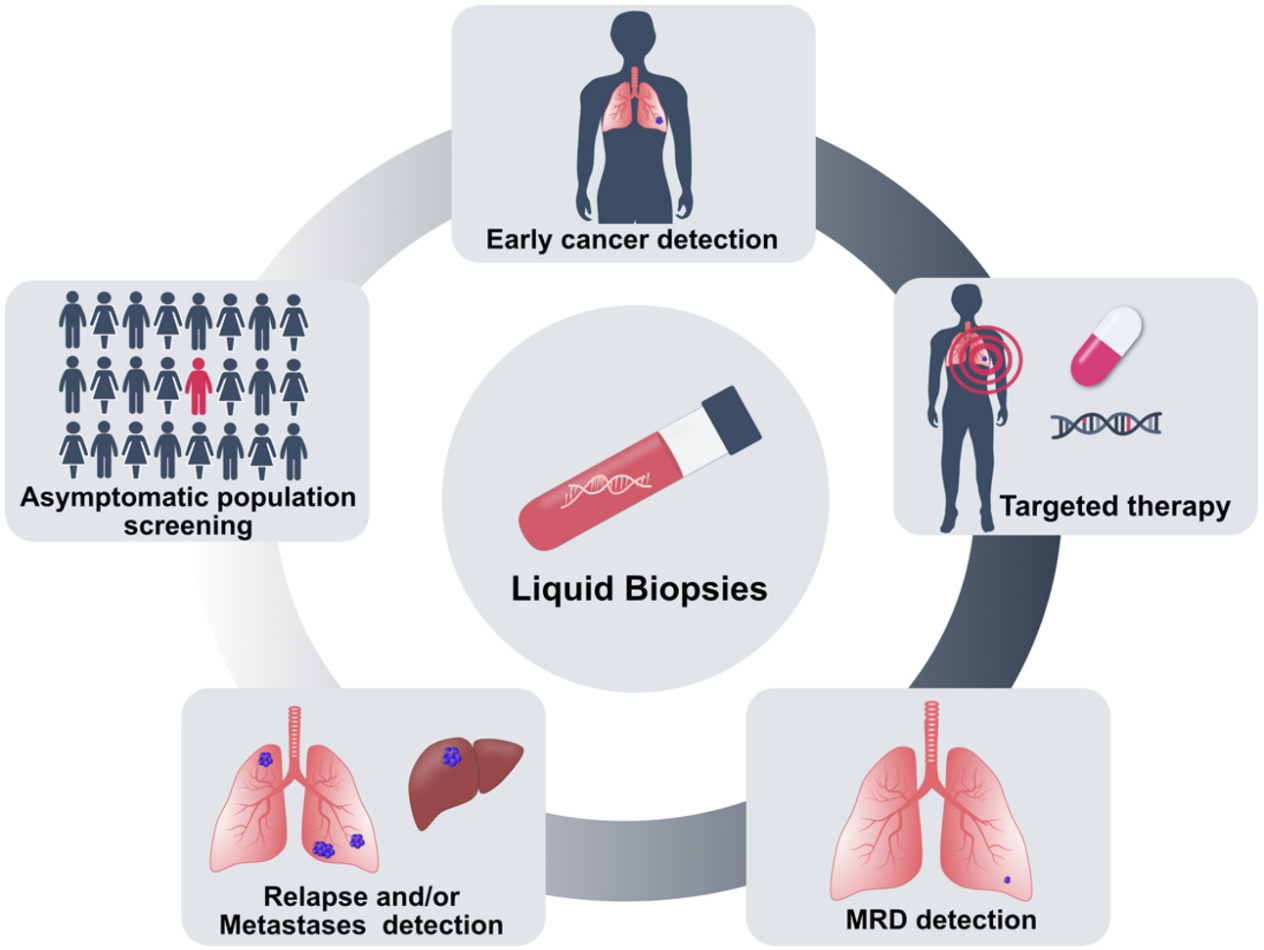
Clinical applications of cfDNA analysis in cancer. Analysis of cfDNA has been used to develop early cancer detection tools and ctDNA mutation analysis can guide therapy choice. After treatment, minimal residual disease (MRD) can be monitored by cfDNA analysis, and in case of resistance, cfDNA-based tests allow detection of relapse and tumor metastases. CfDNA analysis has the potential to screen cancers in the asymptomatic population.

The clinical potential of liquid biopsy for cancer management has been considerably illustrated. Different ctDNA-based biomarkers or assays have been approved by the Food and Drug Administration (FDA). These include two single-cancer detection tests, namely the cobas *EGFR* Mutation Test v2 (Roche Molecular Diagnostics) for lung cancer and Epi proColon (Epigenomics AG) for colorectal cancer, and two targeted NGS-based pan-cancer tests: FoundationOne Liquid CDx (Foundation Medicine) and Guardant360 CDx (Guardant Health), which have been introduced as companion diagnostics to identify biomarkers in patients with advanced diseases. The noninvasive nature of cfDNA allows routine analysis of tumors that are unsafe or infeasible to biopsy [Figure 6]. Moreover, cfDNA may better reflect tumor heterogeneity harbored by distinct clonal populations. Therapeutic guidance is among the most clinically relevant current utilization of ctDNA, particularly for patients for whom standard tumor biopsies fail to yield sufficient material for analysis^[121,122]^. Studies have demonstrated that ctDNA testing may reveal actionable genomic mutations to guide treatment decisions^[123,124]^. In addition, liquid biopsy provides a way to monitor tumor burden and the development of therapy resistance in real-time. It has been shown that levels of patient-specific tumor alterations in cfDNA correlate with the overall tumor burden through serial ctDNA testing^[67,125,126]^. Biological resistance can be a consequence of tumor heterogeneity. ctDNA monitoring permits detection of secondary mutations associated with treatment resistance^[52,127,128]^. Furthermore, the longitudinal analysis of ctDNA may be extended after completion of treatment and may also serve as a means of detecting postsurgical minimal residual disease (MRD) in patients with clinically undetectable disease. Several groups have explored using postoperative ctDNA to identify patients with MRD and continued to optimize technologies^[79,129,130]^. A fraction of malignancies is associated with human viral infection. For the infection-induced malignancies, such as nasopharyngeal carcinoma and oropharyngeal cancer with viral DNA integrated into the host genome, plasma viral cfDNA can be quantified. Monitoring the viral cfDNA has shown prognostic potential for MRD detection^[131,132]^. One of the most impactful applications of ctDNA could be the potential for early cancer detection in asymptomatic populations. Early detection of cancer could increase the chances of survival. While governmental screening programs are in place for some of the cancers (e.g., breast), for many cancers, there are no screening tests available. One study evaluated analysis of Epstein-Barr virus DNA in plasma samples to screen nasopharyngeal carcinoma in asymptomatic populations, and it revealed higher positive predictive value than existing blood-based markers. A significantly higher proportion of participants with the early stage disease was identified, demonstrating the potential for early detection and early treatment intervention^[133]^. By far the biggest commercially driven endeavour for multi-cancer detection, the Galleri^TM^ test, was developed by the company GRAIL^[87,134]^. The test was developed based on 50 different cancer types, and large-scale trials are currently ongoing to evaluate its performance for cancer screening. The combined test that uses multiparameter or multianalyte has also emerged as a key player in cancer detection^[135-137]^. The feasibility and safety of liquid biopsy for multi-cancer screening was largely addressed by a pioneering large clinical trial study using the DETECT-A test which combined ctDNA and protein biomarkers and performed positron emission tomography-computed tomography in patients with a positive blood test^[138]^. In addition to the general population, several studies have reported incidental findings of maternal malignancies during routine NIPS^[139,140]^. These findings have prompted large-scale or nationwide evaluations of occult maternal malignancy detection following abnormal NIPS profiles and emphasized the need to establish multidisciplinary care for clinical management of cancer in pregnancy^[141,142]^. Additionally, the utility of the NIPS platform - analyzing plasma cfDNA with low-pass whole-genome sequencing - for cancer detection has also been explored in high risk asymptomatic populations^[74]^.

### Other applications for cfDNA analysis

The increasing interest in the use of cfDNA is not just within obstetrics and oncology. Transplantation and infectious diseases are active research areas. Based on the rationale that graft rejection entails injury and leads to increased cell death in the allograft, donor-derived cell-free DNA (dd-cfDNA) as a noninvasive marker of graft rejection has been established in solid organ and bone marrow transplants^[143-145]^. Quantifying the concentration of total cfDNA after transplantation, as well as the proportion of the dd-cfDNA subfraction using genotypes, allows detection of allograft injury and immunosuppression. Methylome profiling of cfDNA has also been shown to capture tissue damage in the kidney allograft^[146]^. Serial dd-cfDNA monitoring can provide timely views of transplant health and immunosuppressive drug effects^[147]^.

Infection can be another major complication affecting the health of patients receiving allograft transplantation. A number of assays that concurrently monitor posttransplant rejection and infection by sequencing cfDNA have been developed^[146,148,149]^. Coupled with NGS, the identification of pathogen DNA in plasma of patients with sepsis, invasive fungal infection and cytomegalovirus^[150-152]^ has broadened our ability to systematically detect clinically relevant pathogens in a noninvasive way. Viral DNA detection during NIPS has been demonstrated as well^[153,154]^. Blauwkamp and colleagues developed and validated a microbial cfDNA (mcfDNA) sequencing assay - the Karius test that allows detection of a wide range of infections^[155]^. Though this test is not able to detect RNA viruses, it has been used to identify secondary and co-infections in patients with COVID-19^[156]^. While tracing microbial pathogens is feasible, the analytical sensitivity could be limited by the scarcity of mcfDNA against the dominating host background^[157,158]^. Additionally, pathogen DNA interpretation may be complicated by reagents used for processing, sample contamination and organisms of uncertain clinical significance^[158]^.

Virus infection can cause damage to multiple organs and tissues. Deconvoluting the cell type mixtures in cfDNA methylation profiles from COVID-19 patients, studies found elevated cfDNA levels and mapped tissue injuries particular to lung and liver compared with healthy controls or patients with other respiratory infections^[159,160]^. Interestingly, both studies highlighted a change in the hematopoietic cell signal as an informative marker for COVID-19 mortality prediction. While Cheng *et al.* observed an increase signal from erythroblasts (red blood progenitor cells) that may involve anemia and inflammation or dysregulation of erythropoiesis in COVID-19 patients^[159]^, Andargie *et al.* underscored the major role of neutrophils (white blood cells) that might aggravate pulmonary inflammation and erythropoiesis as a second contributor in elevated cfDNA levels^[160]^. These observations may have an implication in other diseases as well. The hallmark of immune-mediated disorders is inflammation. Plasma DNA might act as an index of inflammation and damage and therefore relate to the altered genomic cfDNA seen in some patients.

### “New” components of cfDNA

Along with cell-free nuclear DNA, cell-free mitochondrial DNA (cf-mtDNA) is detectable in plasma and other fluids. While cf-mtDNA research has fallen behind that of cell-free nuclear DNA, recent investigations into mitochondrial function and cf-mtDNA have elucidated its potential biological significance. Mitochondrial DNA (mtDNA) is prone to oxidative stress and may vary from dozens to thousands of copies depending on tissue origin^[161]^. Different forms of mitochondrial DNA, including free, particle-associated and respiratory competent mitochondria, were reported in plasma samples^[162,163]^. Cf-mtDNA copy number did not appear to be correlated with mtDNA copy number in cellular leukocytes, with the former being more sensitive to cellular damage and the latter reflective of cellular energetics^[164]^. Unlike cell-free nuclear DNA, cf-mtDNA size profile lacks nucleosome-size peaks and shows an enrichment of short fragments smaller than 100 bp^[9]^ due to the lack of histones. Though limited by different pre-analytical protocols and mixed conclusions, cf-mtDNA has been found to be elevated in multiple pathologies, including trauma, sepsis, neurological disorders, and cancer^[164-168]^. A highlight of recent cf-mtDNA research comes from the identification of both intact circular and fragmented linear cf-mtDNA in plasma DNA. Of particular interest, the authors observed that non-hematopoietically derived cf-mtDNA was predominantly in a linear form, whereas hematopoietically derived cf-mtDNA was primarily circular using a transplantation model^[169]^. It is noteworthy that analogous observations regarding fetal and maternal cf-mtDNA in surrogate pregnancies have subsequently been reported by the group^[170]^. The fetal-derived cf-mtDNA mainly existed in a linear form, but about half of maternal-derived cf-mtDNA appeared to be in a circular form. Although there is a major knowledge gap between the triggers that connect pathophysiological states to the cf-mtDNA release into circulation and different biological forms of cf-mtDNA, these findings might prompt the development of cf-mtDNA assays.

Another circular DNA - extrachromosomal circular DNA (eccDNA) has been known for a long time. The recent development of an assay to quantify eccDNA in plasma DNA (referred to as cf-eccDNA throughout the text) entices renewed interest. This circular DNA exists as small (200 to 400 bp), non-amplified eccDNA in normal cells^[171]^ and as large (1.29 Mb EGFRvIII were detected), copy number-amplified extrachromosomal circular DNA (referred to as ecDNA to differentiate from eccDNA) that is primarily found in cancer cells^[172]^. Recent studies demonstrated that ecDNA is common in human cancer cells (up to hundreds of ecDNA molecules per cell), with the highest abundance in brain tumors. Support for the presence of cf-eccDNA in circulation was first shown in two studies that enriched circular DNA by exonuclease digestion of the background linear DNA^[173,174]^. These studies demonstrated that cf-eccDNA harbors characteristics of eccDNA that tends to be generated from regions with high GC content, gene density, active chromatin marks and a high frequency of direct repeats flanking eccDNA junctions. Furthermore, the size of cf-eccDNA tended to be longer when comparing pre- to post-surgery in lung and ovarian cancer patients, possibly implying that cancer-associated eccDNAs are longer than the normal counterparts^[173]^. It has been found that fetal-derived eccDNA is present in the plasma of pregnant women. The cf-eccDNA fragment size distribution was shown to have a small peak at 202 bp, another major peak at 338 bp and a 10 bp periodicity in the vicinity of this peak. The fetal cf-eccDNA tended to be shorter than the maternal counterparts, consistent with the phenomenon in linear nuclear cfDNA^[175]^. Reminiscent of nuclear cfDNA patterns, fetal cf-eccDNA shows relatively lower methylation levels than those of the maternal cf-eccDNA and clears rapidly after delivery with a half-life of 30 min. Longer cf-eccDNA fragments appears to carry higher methylation densities in plasma of both pregnant and non-pregnant subjects^[176]^. Furthermore, nuclease activity on cf-eccDNA was reported, showing that DNASE1L3 affected eccDNA characteristics^[177]^.

### New frontiers in cfDNA biology

CfDNA has long been considered as cell death debris. However, growing evidence points to potential functional aspects of cfDNA, including its role in horizontal genetic transfer, cellular signaling, oxidative stress response, and innate immunity^[1,178,179]^. Immunological properties of cfDNA can be particularly relevant to certain disease pathologies. Interplaying with nuclease biology, the presence of endogenous DNase allows proper DNA degradation and prevents inflammatory stimulation, whereas DNase-deficient mice were prone to develop autoimmune pathologies^[180]^ and exhibited cfDNA fragmentation aberrations^[103]^. Indeed, DNase1 activity was found to be substantially lower in patients with systemic lupus erythematosus (SLE)^[181]^. In pathological conditions like lupus, cfDNA complexed with other proteins may act as proinflammatory stimulants of toll-like receptors (TLR) that elicit the inflammatory response^[107]^. Such mechanisms of recognizing extracellular DNA as damage-associated by the innate immune system has been shown in the case of cf-mtDNA in patients with trauma^[165]^. Triggered by the innate immune response, NETosis that initiates the formation of Neutrophil Extracellular Traps (NETs) could promote thrombosis and mediate tissue damage^[182]^. Formed as extracellular web-like structure with antimicrobial proteins, the NETs have been suggested to play a role in the pathogenesis of multiple diseases, including lupus, sepsis and tumor progression^[183-186]^. Remarkably, studies have found that host DNase1 and DNase1L3 act as dual protection systems to degrade NETs and in the absence of DNase1 and DNase1L3, intravascular NETs form clots and result in organ damage^[187]^. The aforementioned studies that map tissue injury in COVID-19 patients may imply potential immunological effects in cfDNA. Additional pertinent evidence reported strong correlation between levels of cfDNA and traditional inflammatory markers, in particular, absolute neutrophil count and myeloperoxidase-DNA that is regarded as a specific marker of NETs in hospitalized COVID-19 patients^[188]^. In transplantation, elevated dd-cfDNA in the presence of allograft injury may indicate its active role in immunological processes as well^[189]^. Rather than showing immunostimulatory characteristics itself, cfDNA may be a non-specific (pro)inflammation and (pro)coagulation marker in numerous inflammatory and cell death pathways. The question is to what extent the immunological effects are reflected in cfDNA and how the changes can be effectively captured.

### Concluding remarks

Fragmented DNA constantly enters our circulation following passive and active release mechanisms. The composition of tissues contributing to cfDNA differs from one disease to another, and across different physiological states. With the rapid maturity of next generation sequencing and bioinformatics analysis, cfDNA analysis has led to robust copy number detection tools in prenatal testing and tumor liquid biopsy. Yet clinical applications lag behind our expanding understanding of cfDNA biology. In particular, epigenetic and fragmentomic characteristics of cfDNA are an untapped source of potential clinical markers in a wide range of diseases. Novel experimental and computational methods capable of simultaneously capturing and integrating cfDNA modalities present attractive opportunities to push analyses further and may allow for synergistic effects. 

## DECLARATIONS

### Copyright

© The Author(s) 2022.
